# Supplementary material for: RATTLE: reference-free reconstruction and quantification of transcriptomes from Nanopore sequencing
Source: Genome Biol. 2022 Jul 8;23:153. doi: 10.1186/s13059-022-02715-w (PMC9264490; doi:10.1186/s13059-022-02715-w)
Supplement: Supplementary file 3 — Additional file 3. Review history. [file 13059_2022_2715_MOESM3_ESM.docx]

**1^st^ round**

**Reviewer 1**

Summary

In this manuscript Rubia el. al. presents a bioinformatic tool called RATTLE which performs reference-free transcript reconstruction from Oxford Nanopore Technology (ONT) long read data. While many methods exist for long-read transcript reconstruction, most well established tools require a reference genome/transcriptome. This means that currently analysis of ONT data for species without a reference genome is limited and/or untested - hence the need for RATTLE. The manuscript is well written and all sub-tasked performed by RATTLE is well benchmarked. The main novelty is that the transcript identification/reconstruction along with a unified easy to use pipeline. The only major comment I have is that the authors need to benchmark RATTLE against other recent tools (or combinations thereof) that claims to solve the same problem. All in all, this is impressive, thorough work. Congratulations.

Revisions needed

Yes - minor.

Major comments

The RATTLE pipeline must be benchmarked with other pipelines that claim to solve the same problem. This includes:

* the RNA-Bloom tool by Nip et. al. 2021 ( https://genome.cshlp.org/content/30/8/1191.full ) which also enables transcript assembly from ONT (and PacBio) data (see their GitHub https://github.com/bcgsc/RNA-Bloom#quick-start-for-long-reads-running ).

* The isONclust + isONcorrect + IsoCon pipeline used by Paul Medvedev and his group. As I understand it the trick is to use the --ignore_ends_len 30 paramter in IsoCon.

I don't believe these need to be included in all benchmark presented in the manuscript. Rather the benchmark should be done only for the final result (aka transcripts identified and quantification accuracy). These tools are probably also worth including for the runtime + memory benchmark.

Minor comments

* For the (human) cancer example described in the abstract/background would a reference (and reference annotation) based approach not be more suitable?

* Background should also mention PacBio data. On the same note is there a good reason why PacBio data not supported?

* Are the implementation ideas all original work or are the ideas (and/or implementation) from previous work? If so that works should be referenced.

* Supplementary figure 1A could be more clear. Currently it looks like a single k-mer from read 1 tun into a the entire read 1 hashing (and read 2 hashing). Maybe use distinct colors for k-mers and hashes? Would it be possible to illustrate two k-mers and how they fit into the hash?

* In regards to the gene sub-clustering:

o Does the variance threshold for the gene to transcript cluster needs optimization per species or dataset (aka should we use different cutoffs when analyzing transcriptomes with varying complexity (aka humans vs C. Elegans.)? If so, how is that done without a reference?

o Is the optimal variance cutoff affected by the number of isoforms per gene? How small changes can you identify. I would like to see some more benchmarking here - and from more than one gene.

* Judging from the github the RATTLE pipeline could also be made into a single script? Would that not be easier for the end users?

* If you want to help readers, you could ensure that all tools (especially RATTLE) is the same color in all figures. Also use proper capitalization in the name of tools throughout the figures - makes it easier to read.

* For figure 2C/D the authors should discuss why is there such a large performance difference between the simulated (2C) and the real data (2D)?

* For Figure 5A.

o Show the individual points (along with) the boxplot.

o I would suggest summarizing the correlations across all datasets (fig 5A + supl fig 9) in the main figure. This could supplement or replace figure 5A?

o I would suggest (also) using spearman correlations to avoid outliers having to large an impact.

* For all violin plots in figure also add some indication of median to make comparisons easier.

* For figure 5C

o Please double-check the plots are not over-smoothed

* Figure 5D: How does the evaluation in figure 5D change as a function of coverage cutoff (the cutoff seems rather arbitrary)? Also use large dot-sizes to make it more viewer friendly.

* For the scalability study:

o I would suggest to also show runtimes on (one of) the real samples (can be down sampled) since as I understand it the simulated samples have very few genes. This would also more relevant for the end users?

o Make y-axis more human readable.

* The authors also need to discuss overcorrection since their method have been shown to be prone to overcorrecting https://www.nature.com/articles/s41467-020-20340-8

Sincerely

Kristoffer Vitting-Seerup

**Reviewer 2**

In this work the authors present RATTLE a software package for reference-free reconstruction and quantification of transcriptomes from Nanopore sequencing data. Overall, the paper is well-written, and the approach is described in appropriate detail. The presented software does not necessarily perform better than other tools in individually tested tasks. However, is manages to perform on par despite not using a reference transcriptome/genome. This feature makes it particularly useful for studying organisms/cells with incomplete annotation or systems that de-novo annotation is required. As Nanopore sequencing is becoming more popular, this software is a great and important addition to the tools that the wider community can use to extract information from long-read sequencing data.

Comments:

1. The authors mention the following as a strength of the software "RATTLE modularity, with the ability to parameterize each step, means it can be easily adapted to any sample type.". I agree flexibility is useful. However, I also think that this is a limitation of the work. In the methods alone, I counted more than 12 parameters that have been selected arbitrarily (e.g. page/line: 16/43, 17/32, 17/39, 18/5, 18/10, 18/23, 18/24, 18/35, 18/41, 18/43). The authors need to provide justification for at least some of these selections and provide some guidance to the user with respect to the software performance upon different choices for these parameters, as for example in Fig. 4c, d. As most users do not typically change the default parameters, ideally and perhaps in a future release, some of these parameters should be extracted directly from the data (for example via an optimization approach) and not be left to the user to tune.

2. In pg 17, ln 9: It is unclear to me why clusters with N reads are separated in blocks. Can the authors elaborate?

3. In pg 4, ln 56-59: I suggest rephrasing to explain why the comparison to the minimap2 minimizer is relevant.

4. Sup. Fig 1b: Table appears to be sorted by read 2, not read1 as described in the legend.

5. In pg 7, ln 16-19: I suggest rephrasing.

6. Fig. 2b: The legend seems to be wrong as there are no lower panels.

7. At which read support are Fig. 4a and b calculated? Is it 5? I would be good to specify in the results so that Fig. 4a, b and Fig. 4c, d can be compared.

8. Fig. 5d: Missing color legend. I assume it's the same as 5e but it's unclear.

**Authors’ response**

Reviewer 1

Summary

In this manuscript Rubia el. al. presents a bioinformatic tool called RATTLE which performs reference-free transcript reconstruction from Oxford Nanopore Technology (ONT) long read data. While many methods exist for long-read transcript reconstruction, most well established tools require a reference genome/transcriptome. This means that currently analysis of ONT data for species without a reference genome is limited and/or untested - hence the need for RATTLE. The manuscript is well written and all sub-tasked performed by RATTLE is well benchmarked. The main novelty is that the transcript identification/reconstruction along with a unified easy to use pipeline. The only major comment I have is that the authors need to benchmark RATTLE against other recent tools (or combinations thereof) that claims to solve the same problem. All in all, this is impressive, thorough work. Congratulations.

We thank the reviewer for the encouraging words and suggestions for improvement.

Revisions needed

Yes - minor.

Major comments

The RATTLE pipeline must be benchmarked with other pipelines that claim to solve the same problem. This includes:

* the RNA-Bloom tool by Nip et. al. 2021 ( https://genome.cshlp.org/content/30/8/1191.full ) which also enables transcript assembly from ONT (and PacBio) data (see their GitHub https://github.com/bcgsc/RNA- Bloom#quick-start-for-long-reads-running ).

We thank the reviewer for this suggestion. We would like to note that RNA-Bloom was published as a tool to operate with Illumina reads. Also, RNA-bloom’s publication (https://genome.cshlp.org/content/30/8/1191.full) does not use long reads. On the other hand, at the time of preparing this revision (15/03/2022), RNA-Bloom’s GitHub claims it works also for long reads but it does not provide any details about its performance and reliability. With this caveat in mind, we have tested RNA-Bloom with our Nanopore data using the instructions from their GitHub page.

We used inputs from different sizes (100k, 250k, 500k, 750k, 1M, 2M, and 3M reads) obtained by subsampling direct-RNA sequencing reads obtained from HEK293 WT cells (https://pubmed.ncbi.nlm.nih.gov/34282325/) (European Nucleotide Archive (ENA) accession PRJEB40872) and run RATTLE and RNA-bloom on each of the subsets independently.

We compared the length distributions of the predicted transcripts (similar to Fig. 5b). The results show that RATTLE transcript lengths are longer and converge faster to the expected length of the annotation as we use an increasing number of input reads. In contrast, RNA-bloom produces much shorter transcripts: In this analysis, the annotation (“ref” in the plots) corresponds to the annotated transcripts (Gencode v29) that show expression after mapping all the 3M reads to the transcriptome with minimap2, keeping the best match for each read. To further compare both tools, we calculated the number of transcripts produced by both methods for each input size (similar to Fig. 5e):

The left panel shows the number of transcripts produced for increasing numbers of input reads (similar to Fig. 5e, left panel). The plot shows that RATTLE tends to saturate for an increasing number of input reads, whereas RNA-bloom produces more and more transcripts. This data is included in Supp. Table S12. The right panel shows the unique number of annotated transcripts recovered by mapping the predicted transcripts to the transcript annotation with minimap2, keeping the best match for each annotated transcript and using the matches with coverage above 75% (similar to Fig. 5e, right panel). This plot shows that RNA-bloom recovers more unique transcripts, but increases more rapidly than RATTLE, whereas RATTLE shows a tendency to saturate in the number of transcripts recovered.

We also compared the memory usage and running times for both tools. This is now included in Supp. Table S12. Although RNA-bloom was faster, it tended to use more memory.

Finally, it is important to highlight that transcript quantification is not documented yet in RNA-Bloom’s GitHub page and does not seem to be yet available from the RNA-Bloom’s output. So we could not benchmark this aspect with RNA-bloom.

In the manuscript, we already include a benchmarking of abundance using several tools (e.g. StringTie2, FLAIR, TALON in Fig 5). As these tools are reference-based, they are expected to provide already an optimal benchmark of the best abundance estimates that can be obtained from Nanopore reads in general.

* The isONclust + isONcorrect + IsoCon pipeline used by Paul Medvedev and his group. As I understand it the trick is to use the --ignore_ends_len 30 paramter in IsoCon. I don't believe these need to be included in all benchmark presented in the manuscript. Rather the benchmark should be done only for the final result (aka transcripts identified and quantification accuracy).

We thank the reviewer for this suggestion. At the time of preparing this revision (15/03/2022), we have not been able to find any example, documentation, or paper describing the suggested combination “isONclust + isONcorrect + IsoCon”.

As described in the IsoCon github (https://github.com/ksahlin/IsoCon), IsoCon’s applicability for long-reads is restricted to “datasets where the sequences span the regions of interest end-to-end”. For Nanopore, it is described as “only applicable for targeted approaches, i.e., amplicon sequencing or Rolling Circle Amplification to Concatermirc Consensus (R2C2)”. None of these criteria apply to the type of datasets used in our manuscript. Thus, IsoCon is not an appropriate tool to apply on unbiased transcriptome-wide nanopore sequencing datasets, such as those used in our manuscript.

The author of these tools also indicated to someone trying to use IsoCon on targeted nanopore sequencing reads (see https://github.com/ksahlin/IsoCon/issues/8) that although “IsoCon --ignore_ends_len 30” may mitigate some of the constraints related to the “end-to-end condition” in the IsoCon algorithm, “IsoCon is currently only tuned for Iso-Seq error profiles”. That is, IsoCon is a tool specifically tuned for Iso-Seq (PacBio) reads. This is a comment from June 2020 and the code in the IsoCon repository has not been updated for the last 2-4 years (last check on 15/03/2022). Thus, we can discard the possibility that this aspect of the tool has been changed.

Furthermore, in the same comment (https://github.com/ksahlin/IsoCon/issues/8), IsoCon’s author suggested “isONclust + isONcorrect” as an alternative to IsoCon, but not in combination with it. We have not identified any other information about the combination of these tools. It is also not clear that this could be a viable combination of tools for the purpose of benchmarking the reconstruction of transcripts from unbiased RNA/cDNA Nanopore sequencing experiments since isONclust produces gene clusters, not transcript clusters.

Finally, we already show in the manuscript comparisons of RATTLE with isONclust (Figs. 2c and 2d, and Supp Tables S2 and S3) and with isONcorrect (Figs 3 and 4, Supp Tables S4, S5, S6, and S7). Thus, we have already used and benchmarked isONclust and isONcorrect for the tasks they were defined to do.

These tools are probably also worth including for the runtime + memory benchmark.

We have tested the maximum CPU memory usage and run times for RATTLE and RNA-Bloom using inputs of different sizes (100k, 250k, ..., 3M reads). RATTLE generally shows longer runtimes but lower memory usage. These results are provided in Supp. Table S12.

Minor comments

* For the (human) cancer example described in the abstract/background would a reference (and reference annotation) based approach not be more suitable?

The current reference is 70% sourced from a single individual and mostly represents European ancestry, which is just ~10% of the world population (https://pubmed.ncbi.nlm.nih.gov/31399121/). Most of the population and cancer variation is described in terms of single nucleotide variants and small indels relative to this reference. Reference-based cancer transcriptomics assumes that cancer alterations can still be matched to reference sequences, including indels, copy number variants, fusions, etc. However, there may be variations that are not detectable in the DNA reference. These include complex rearrangements, expression from polymorphic mobile elements or viral insertions that are specific to an individual or population, and expression from genetic sequences that are either divergent from the reference or not yet included in the reference assembly. These cases might be particularly relevant in germline and somatic genomes from ancestries not represented in the reference and highly divergent from it. Reference-free transcriptomics provides an additional tool to explore and identify transcripts that could have been missed by current reference-based methods.

* Background should also mention PacBio data. On the same note is there a good reason why PacBio data not supported?

At the start of the project we did not have as many PacBio datasets available as we had for Nanopore, and we had the possibility to generate our own Nanopore data, which we have deposited in the European Nucleotide Archive (study PRJEB40410). We thus decided to focus on Nanopore, which presents additional challenges to PacBio. These include the higher error rate compared to PacBio, longer lengths and wider length distribution, and a higher error rate of the direct RNA reads compared to the cDNA-based protocols. Addtionally, since Nanopore read lengths are theoretically unbounded, unlike PacBio data, we considered they would provide a good opportunity to test the reconstruction of full transcripts without a reference which, to the best of our knowledge, has not been tested yet.

* Are the implementation ideas all original work or are the ideas (and/or implementation) from previous work? If so that works should be referenced.

All methods and ideas from previous works have been referenced.

* Supplementary figure 1A could be more clear. Currently it looks like a single k-mer from read 1 tun into a the entire read 1 hashing (and read 2 hashing). Maybe use distinct colors for k-mers and hashes? Would it be possible to illustrate two k-mers and how they fit into the hash?

Thanks for pointing this out. We have updated the plot to make it more clear. We provide here a more extended explanation:

Given a read >read1

ACATGGAGGTATTTTGACATCTAAGGTATT

Each k-mer in a read is represented as a binary number using A=00, C=01, T=11, G=10 and then recoded by the hash. For the underlined 6-mer:

ACATGG → 000100111010 → Hash (000100111010) = 314.

This will set the position 314 of read1 k-mer vector to 1.

So the read1 k-mer vector will have 1 in the positions given by the hash for all k-mers extracted from the read, and 0 for all other positions. Note that if a k-mer appears multiple times, they all have the same position, and this is only recorded once.

For a second read this is done similarly, e.g. >read2

ACATAGTTGGTATTAGCTGATCGGTCGAAC ACATAG → Hash(000100110010) = 306 → set read2 kmers vector position 306 to 1

The first RATTLE metric makes then a comparison of the two k-mer vectors for read1 and read2 using an AND operation:

⇓

0010011000000...010 ← read1 k-mer vector

0100010011100...010 ← read2 k-mer vector

⇓

0000010000000...010 ← AND operation

* In regards to the gene sub-clustering:

o Does the variance threshold for the gene to transcript cluster needs optimization per species or dataset (aka should we use different cutoffs when analyzing transcriptomes with varying complexity (aka humans vs C. Elegans.)? If so, how is that done without a reference?

The variance is a parameter that can be set by the user:

--iso-max-variance = max allowed variance for two reads to be in the same isoform cluster (default: 25)

This value is calculated as the variance of the distribution of the relative differences in length between adjacent matching k-mers between two reads in a gene-cluster. These k-mers are defined by this parameter:

--iso-kmer-size = k-mer size for isoform clustering (default: 11)

The variance threshold then indicates the value above which, this variance is expected to be due to differences in exons rather than errors. As this variation is due to the errors in the reads, it does not depend on the species, but on the error-rate of the reads and can be considered a generic parameter. On the other hand, the length of the alternative exons that can be detected will depend on the value of this parameter (see analysis below). These lengths may be different across species, in which case the parameter needs to be modified.

o Is the optimal variance cutoff affected by the number of isoforms per gene? How small changes can you identify. I would like to see some more benchmarking here - and from more than one gene.

To better illustrate the effect of the --iso-max-variance parameter and the isoform differences that RATTLE can detect, we have performed a systematic comparison using reads simulated from pairs of isoforms that differ by an exon of variable length.

We analysed three pairs of isoform transcripts: pair 1 (ENST00000378307.9, ENST00000357052.8), pair 2

(ENST00000667721.2, ENST00000684870.1), pair 3(ENST00000525981.1, ENST00000526562.5). Each pair of isoforms differed from each other by one internal alternative exon (>150nt). We removed sequence from the alternative exon to obtain 10 variable exon lengths between 25 and 150 (25, 50, 55, 60, 65, 70, 75, 100, 125, 150). We used DeepSimulator to simulate 1000 Nanopore RNA reads from each isoform. Then, we ran RATTLE and only changed --iso-max-variance parameter value from 5 to 100 (5, 10, 15, 20, 25, 50, 100). That is, we ran RATTLE for 10 x 7 = 70 different configurations for each isoform pair. For all other parameters, we used the default values provided on the GitHub page (https://github.com/comprna/RATTLE).

For all simulations, each corresponding to an alternative exon length and a –max-iso-variance value, RATTLE identified 1 single gene cluster. This is in agreement with the good accuracy shown by RATTLE to obtain gene clusters as shown in the manuscript (Fig. 2, Supp Tables S2, S3). For each simulation, we further calculated the number of transcript clusters produced and the abundance of the most abundant transcript cluster. The abundance was calculated as the proportion of reads from the gene cluster that supports a given transcript. A perfect result would correspond to two transcript clusters and an abundance of 50% for both of them.

We found that RATTLE can recover the correct clusters from exon length >= 50 with --iso-max-variance >=5.

For higher values of --iso-max-variance, the minimal exon length that can be recovered increases. The three plots below show on the left panels the number of transcript clusters (y-axis) at different exon lengths (x-axis) using different --iso-max-variance (colour).

We also found that RATTLE can recover the correct transcript abundances from exon length >= 65 with --iso-

max-variance >=5. The plots show on the right panels the abundance of the most abundant transcripts (y-axis) (expressed as the percentage of reads in the gene cluster) for each exon length (x-axis) and for each --iso-max- variance (colour) values as the left ones. Pairs 1 and 2 have similar transcripts lengths (~1000nt), and the third pair is much shorter (~300nt). The results from pair 3 show a better recovery of the isoform abundances, suggesting that shorter transcripts might be easier to separate.

These plots are now included as Supp. Figure 2, and the data is available in Supp. Table S1.

* Judging from the github the RATTLE pipeline could also be made into a single script? Would that not be easier for the end users?

The three RATTLE steps can be easily integrated into a single pipeline. We have added a snakemake pipeline in RATTLE’s github (https://github.com/comprna/rattle#Snakemake) that integrates the three steps. RATTLE’s modularity presents multiple advantages that do not hinder RATTLE’s ease of use:

● Each of the operations can be run separately or in combination with other tools. For instance, our analyses showed that for the clustering step, RATTLE is much more efficient than the other tools. One could then build clusters with RATTLE and process them further with other tools if desired.

● One can run each of the steps multiple times with different parameters to assess the outputs, before proceeding to the next step.

● RATTLE’s modularity makes it easier to extend the software capabilities. For instance, extending RATTLE to run on multiple samples would enable performing differential expression analysis between conditions simply using clusters, without having to run the other steps.

* If you want to help readers, you could ensure that all tools (especially RATTLE) is the same color in all figures. Also use proper capitalization in the name of tools throughout the figures - makes it easier to read.

Thanks for pointing this out. We were aware of the relevance of the consistency of colors and had already made sure that this was observed throughout the manuscript:

● Tools in Fig 2 have consistent colours, with no tools in common with the other figures. RATTLE’s different colour from the other figures is justified by the fact that it refers only to the clustering step.

● Tools in Figs 3 and 4 have consistent colours for the tools across all panels in these two figures, with no tools in common with the other figures. RATTLE’s color is consistent in Figs 3 and 4 and it refers only to the error-correction step.

● All tools in the panels of Figure 5 have consistent colors, with no tools in common with the other figures. RATTLE’s color is consistent in Fig 5 and refers only to the output of the final transcript reconstruction and polishing step.

We have added capitalisation of the tool names in the main figures (Fig. 3, Fig. 4, Fig. 5). We have changed the legends accordingly.

* For figure 2C/D the authors should discuss why is there such a large performance difference between the simulated (2C) and the real data (2D)?

Thanks for pointing out this difference. The simulated data is more homogeneous as, in each dataset, we simulated the same number of reads per transcript, even though we were using different numbers of transcripts per gene. We would expect then that errors impact in a similar way all genes and transcripts.

In contrast, SIRV read data is more heterogeneous. First, SIRV transcript relative abundances vary several orders of magnitude within each sample (please see Supp. Table S8 for the relative abundances of the SIRV isoforms). As a consequence, the variability in the number of reads available for each SIRV transcript will be larger. Second, SIRV isoform sequences overlap quite significantly with each other and some of them are fully overlapping in opposite strands (see https://www.lexogen.com/sirvs/design/). This makes it very hard to separate the SIRV reads into different clusters. In particular, for Nanopore cDNA-seq, unless the adapters are stranded or reads re-oriented, reads from fully overlapping isoforms in opposite strands cannot be separated. Some methods will be more sensitive to the variability of the SIRV data, resulting in worse accuracies. As we show in the Supp. Table S3, CARNAC tends to produce worse values for completeness than for homogeneity. Homogeneity is maximal when each cluster contains only elements of the same class, whereas completeness is maximal when all the elements of a single class are in the same cluster. This indicates that CARNAC tends to mix reads from different SIRVs in the same cluster.

We have added a comment regarding this topic in the manuscript.

* For Figure 5A.

o Show the individual points (along with) the boxplot.

The data points for these plots are available in Supp. Table S8

o I would suggest summarizing the correlations across all datasets (fig 5A + supl fig 9) in the main figure. This could supplement or replace figure 5A?

Thank you for this suggestion. After carefully considering this suggestion, we have finally decided to keep this supplementary figure (now Supp. Fig. 10) with the additional datasets separated from the main figure, as the information is quite redundant. The original data points for all these comparisons are available in Supp. Table S8.

o I would suggest (also) using spearman correlations to avoid outliers having to large an impact.

Thanks for this suggestion. We have added the Spearman correlations to Supplementary Table S8, together with the Pearson correlation values already present in the Figures. The overall conclusions do not change:

RATTLE performs similarly to reference-based methods with cDNA and RNA.

* For all violin plots in figure also add some indication of median to make comparisons easier.

In most Figures, we have used box plots and indicated the median. In the case of the violin plots in Figure 5c the main point was to show that the distribution was bimodal and the median would not be informative. In Figure 5b, we are showing the median from the reference transcriptome, and the figure already clearly shows how all distributions are below this median.

* For figure 5C

o Please double-check the plots are not over-smoothed

Thank you for raising this potential issue. We checked and the plots are not over-smoothed.

* Figure 5D: How does the evaluation in figure 5D change as a function of coverage cutoff (the cutoff seems rather arbitrary)? Also use large dot-sizes to make it more viewer friendly.

The transcript length coverage distributions (Fig 5c) show bimodality, and 75% approximately indicates the separation of these two main groups. 75% provides a cutoff for the confidence in assigning a transcript prediction to its most likely corresponding transcript annotation. We repeated the analysis at different coverage cutoffs. The correlation of the abundances, with cDNA or dRNA reads, is quite stable across all tools:

We have added this figure as Supp. Fig. 13.

* For the scalability study:

o I would suggest to also show runtimes on (one of) the real samples (can be down sampled) since as I understand it the simulated samples have very few genes. This would also more relevant for the end users?

We have updated Fig. 6a and 6b using a set of >3M reads from Hek293 WT cells from https://pubmed.ncbi.nlm.nih.gov/34282325/ (European Nucleotide Archive (ENA), accession PRJEB40872) and downsampling to 100k, 250k, 500k, 750k, 1M, 2M, and 3M reads. The complete scaling information with these datasets is available in the Supp. Table S12.

o Make y-axis more human readable.

Thank you for this suggestion. We have now updated Figs 6a and 6b accordingly.

* The authors also need to discuss overcorrection since their method have been shown to be prone to overcorrecting https://www.nature.com/articles/s41467-020-20340-8

RATTLE corrects the reads within transcript clusters. So reads corresponding to different isoforms are not expected to be lost because of overcorrection, but rather due to the assignment to the wrong transcript cluster. So this point may have been a misunderstanding by that publication. We have now added an additional benchmarking analysis of the separation of reads into transcripts (described above, add as Supp. Fig 2 and Supp Table S1). This analysis shows that when RATTLE misses a transcript isoform or does not predict the abundance correctly is because reads are assigned to the wrong transcript cluster. We have added a comment on this in the results and discussion sections.

Sincerely

Kristoffer Vitting-Seerup

Reviewer #2: In this work the authors present RATTLE a software package for reference-free reconstruction and quantification of transcriptomes from Nanopore sequencing data. Overall, the paper is well-written, and the approach is described in appropriate detail. The presented software does not necessarily perform better than other tools in individually tested tasks. However, is manages to perform on par despite not using a reference transcriptome/genome. This feature makes it particularly useful for studying organisms/cells with incomplete annotation or systems that de-novo annotation is required. As Nanopore sequencing is becoming more popular, this software is a great and important addition to the tools that the wider community can use to extract information from long-read sequencing data.

We thank the reviewer for the supporting words. We have compared RATTLE’s individual operations with tools specific to each of these operations. We showed that for clustering and error correction RATTLE performs better or is comparable to other reference-free methods. Due to the overall lack of reference-free tools for transcript reconstruction and quantification with Nanopore reads, we performed comparisons with reference- based methods, which have the obvious advantage of using the genome reference, but provide the maximum possible accuracy that can be attained with Nanopore reads. For reference-free transcript reconstruction, we have now added additional comparisons with RNA-Bloom, which is not published or preprinted, but is available from GitHub (https://github.com/bcgsc/RNA-Bloom/).

Comments:

1. The authors mention the following as a strength of the software "RATTLE modularity, with the ability to parameterize each step, means it can be easily adapted to any sample type.". I agree flexibility is useful. However, I also think that this is a limitation of the work. In the methods alone, I counted more than 12 parameters that have been selected arbitrarily (e.g. page/line: 16/43, 17/32, 17/39, 18/5, 18/10, 18/23, 18/24, 18/35, 18/41, 18/43). The authors need to provide justification for at least some of these selections and provide some guidance to the user with respect to the software performance upon different choices for these parameters, as for example in Fig. 4c, d. As most users do not typically change the default parameters, ideally and perhaps in a future release, some of these parameters should be extracted directly from the data (for example via an

optimization approach) and not be left to the user to tune.

We thank the reviewer for pointing this out. All the parameters, their definitions, and the default values are described in RATTLE’s GitHub https://github.com/comprna/RATTLE In the README we also indicate the maximum values allowed, which are determined by the data structures used in the RATTLE algorithms.

In the Supp. Table S11, we provide the memory footprint and accuracy for different parameter variations compared with the default values. We have added details on these results and comparisons in the manuscript (highlighted in blue). These parameters are probably the most important ones in terms of the speed and the accuracy of the clustering of reads into genes:

-B (--bv-start-threshold) = the starting threshold for the bitvector k-mer comparison (default: 0.4). This bitvector kmer comparison is described in supp. Fig. 1a.

-b (--bv-end-threshold) = the ending threshold for the bitvector k-mer comparison (default: 0.2). The greedy clustering is performed iteratively by shifting the threshold of the bitvector comparison from -B to -b.

-f (--bv-falloff) = the falloff value for the bitvector threshold for each iteration (default: 0.05). This is the stride or falloff value used to shift from -B to -b.

We have added a benchmarking of the Other relevant parameters, not included in those tests, are the different k-mer sizes used:

-k (--kmer-size) (default = 10) = k-mer size chosen for the pairwise comparison of reads through the bitvector. A larger k here will speed up the clustering, but will lead to worse clustering results, due to the high frequency of errors in Nanopore reads.

--iso-kmer-size (default = 11) = k-mer size for determining the separation of reads in a gene- cluster into transcript-clusters. This k-mers are used to compare pairs of reads within the same gene cluster.

This is expected to be similar to -k above, to avoid separating reads into different transcript-clusters due to errors rather than differences in exonic sequence content.

We now also provide an additional benchmarking of the parameter

--iso-max-variance

max allowed variance for two reads to be in the same isoform cluster (default: 25)

These analysis indicate that although the default given is 25, a lower value might be convenient to detect variations due to small exons (<70nt).

Besides the input parameters, there are the output features. Figure 4c and 4d show output features rather than RATTLE input parameters. They provide a critical assessment of how the accuracy of the outputs from RATTLE and other tools depends on the read support. This analysis looks critically at the support of the transcripts and points out a large number of false positives if the read-support is not considered. This is a relevant outcome from our paper and is generally lacking in the related literature. This analysis was performed with the default parameters. The reviewer’s suggestion is an excellent idea to provide an optimization of other parameters in a future release.

2. In pg 17, ln 9: It is unclear to me why clusters with N reads are separated in blocks. Can the authors elaborate?

This is related to RATTLE’s error-correction algorithm (page 18, line 9). The algorithm is based on a multiple sequence alignment (MSA) of the reads within a transcript cluster. To mitigate the computational costs and avoid the artefacts related to performing an MSA with many sequences (see e.g. https://pubmed.ncbi.nlm.nih.gov/23428640/), we devised the following heuristics in RATTLE. All the reads in the cluster are separated into subsets that we call blocks. Blocks are built in such a way that the distribution of read lengths is balanced across blocks. RATTLE performs the MSAs using Partial Order Graphs (POGs) built with SPOA (https://github.com/rvaser/spoa). POGs are sensitive to the read lengths and the order in which the reads are used may impact the results. The methods section describes how this splitting is made so that each block contains similar length distributions. Each block is sorted in descending order according to length so that the first read to build a POG is always the longest. Each block is then used to build an MSA and a consensus sequence is derived from the MSA. The consensus sequences from all the blocks are then processed again with a POG to build a new MSA, which is used to obtain the final consensus sequence. The block partitioning makes this much more computationally efficient and avoids the artefacts that are introduced when attempting to obtain an MSA with many sequences.

3. In pg 4, ln 56-59: I suggest rephrasing to explain why the comparison to the minimap2 minimizer is relevant.

Thanks for this suggestion.

Minimap2 uses minimizers to calculate the matching scores between two sequences. A minimizer is defined to be the first k-mer in a list of all the k-mers from a given sequence (length-k substrings) that have been alphabetically sorted (for a fixed k). Running a sliding window through a read, one can define a minimizer for each window. Usually, the minimizer stays the same for multiple overlapping windows. This property makes the comparison between reads using minimizers very computationally efficient. RATTLE compromises efficiency for accuracy. RATTLE uses first a hashing of 6-mers for a quick approximate match, and then a longest increasing subsequence (LIS) algorithm to confirm matches pairwise. Fig 2a shows that this matching algorithm can be more accurate than the matching score from Minimap2 based on minimizers. This is relevant to building accurate read clusters in a reference-free approach. Minimizers will not be problematic in reference- based approaches in general, as they are used to match multiple reads to a single high-accurate reference. We have now changed the text to clarify why this comparison is relevant:

RATTLE similarity score separated reads originating from different transcripts better than using the score based on a sequence alignment using Minimap2 [16], which is based on minimizers (Fig. 2a). This indicates that although minimizers provide an efficient comparison between sequences, RATTLE similarity score may be more robust for comparisons between error-prone reads.

4. Sup. Fig 1b: Table appears to be sorted by read 2, not read1 as described in the legend.

Thanks for pointing this out. We have fixed the Supp Fig 1b to agree with the description of the algorithm:

(ACAT, 0, 0)

(GGTA, 7, 7)

(GTAT, 8, 8)

(TATT, 9, 3)

(TATT, 9, 9)

We have also added a match in position 8, which was missing.

5. In pg 7, ln 16-19: I suggest rephrasing.

Thank you for pointing out the lack of clarity. We have rephrased the text as follows:

Both RATTLE and isONcorrect achieved results close to TranscriptClean, which had the best improvement over raw reads. TranscriptClean results were similar for cDNA and dRNA reads. However, self-correction with dRNA reads did not improve percentage identity and decrease error-rate as much as with cDNA reads.

6. Fig. 2b: The legend seems to be wrong as there are no lower panels.

We apologise for the confusion. We have fixed the figure legend.

This refers to the Fig. 3b legend, which now reads:

Figure 3. We show the percentage identity distributions of all SIRV reads before (raw) and after correcting with RATTLE, CONSENT, isONcorrect, Canu, and TranscriptClean (clean) for Nanopore cDNA (sample cDNA2) (a) and dRNA reads (sample RNA1) (b). Percentage identity was calculated as the number of nucleotide matches divided by the total length of the aligned region. Other samples are shown in Supp. Fig. 3. We also show the error rate distribution of SIRV reads before (raw) and after correction with RATTLE, CONSENT, isONcorrect, Canu, and TranscriptClean (clean) for the same cDNA (c) and RNA (d) samples. The error rate was calculated as the sum of insertions, deletions, and substitutions divided by the length of the read. Other samples are shown in Supp. Fig. 4. Finally, we show the distribution of percentage identity (e) and error rate (f) for reads from the human transcriptome before (raw) and after correcting with RATTLE, CONSENT, isONcorrect, and TranscriptClean (clean) for Human heart dRNA-seq (RNA1). Percentage identity and error rate was calculated as in (a-d).

7. At which read support are Fig. 4a and b calculated? Is it 5? I would be good to specify in the results so that Fig. 4a, b and Fig. 4c, d can be compared.

Thanks for pointing this out. Fig. 4a and 4b were calculated for read support > 5. We have now added this to the figure legend.

8. Fig. 5d: Missing color legend. I assume it's the same as 5e but it's unclear.

Thanks for pointing this out. Indeed, the colour used for each method is the same throughout Figs. 5b, c, d, e. We have indicated this in the Figure caption.

**2^nd^ round**

**Reviewer 1**

I just want to congratulate the authors on their very thorough work and revisions.

**Reviewer 2**

The authors have addressed my comments at a satisfactory level.

As a side note, I would encourage the authors to simplify the tool parameters and provide more guidance to the users as to how to tune them. Looking at the Github page, many of the parameters are quite cryptic, unless the user dives in the paper figures and fully understands the algorithm.

For example:

-s, --score-threshold

minimum score for two reads to be in the same gene cluster (default: 0.2)

A user does not know what the score is, how it's calculated or if 0.2 is a large or small number to tune it accordingly.
